# Senescence-associated exosomes transfer miRNA-induced fibrosis to neighboring cells

**DOI:** 10.18632/aging.204539

**Published:** 2023-02-23

**Authors:** Amy H. Lee, Deepraj Ghosh, Ivy L. Koh, Michelle R. Dawson

**Affiliations:** 1 School of Engineering, Center for Biomedical Engineering, Brown University, Providence, RI 02912, USA; 2 Molecular Biology, Cell Biology, and Biochemistry, Brown University, Providence, RI 02912, USA

**Keywords:** radiation-induced senescence, exosomes (EXOs), microRNA (miRNA), transforming growth factor-β (TGF-β), mesenchymal stem cells (MSCs)

## Abstract

Radiation-induced fibrosis is a common side effect of radiotherapy, which is the most common treatment for cancer. However, radiation also causes p53-mediated cell cycle arrest, prolonged expression of p21, and the development of senescence in normal cells that reside in irradiated tissues. Bone marrow-derived mesenchymal stem cells (MSCs) accumulate in primary tumor sites because of their natural tropism for inflammatory and fibrotic tissues. MSCs are extremely sensitive to low doses of ionizing radiation and acquire senescence as a result of bystander radiation effects. Senescent cells remain metabolically active but develop a potent senescence-associated secretory phenotype (SASP) that correlates to hyperactive secretion of cytokines, pro-fibrotic growth factors, and exosomes (EXOs). Integrative pathway analysis highlighted that radiation-induced senescence significantly enriched cell-cycle, extracellular matrix, transforming growth factor-β (TGF-β) signaling, and vesicle-mediated transport genes in MSCs. EXOs are cell-secreted nanovesicles (a subclass of small extracellular vesicles) that contain biomaterials—proteins, RNAs, microRNAs (miRNAs)—that are critical in cell-cell communication. miRNA content analysis of secreted EXOs further revealed that radiation-induced senescence uniquely altered miRNA profiles. In fact, several of the standout miRNAs directly targeted TGF-β or downstream genes. To examine bystander effects of radiation-induced senescence, we further treated normal MSCs with senescence-associated EXOs (SA-EXOs). These modulated genes related to TGF-β pathway and elevated both alpha smooth muscle actin (protein increased in senescent, activated cells) and Ki-67 (proliferative marker) expression in SA-EXO treated MSCs compared to untreated MSCs. We revealed SA-EXOs possess unique miRNA content that influence myofibroblast phenotypes via TGF-β pathway activation. This highlights that SA-EXOs are potent SASP factors that play a large role in cancer-related fibrosis.

## INTRODUCTION

Cancer treatment with radiotherapy is highly effective in killing cancer cells by inducing DNA damage and apoptosis; however, ionizing radiation damages DNA in normal cells inducing senescence [1]. Exposure to ionizing radiation also results in rapid activation and persistent expression of transforming growth factor-β (TGF-β)—a pleiotropic cytokine involved in extracellular matrix (ECM) remodeling [2]. Accumulation of senescent cells and persistent activation of TGF-β have been implicated in tissue fibrosis. Senescent cells remain metabolically active but are often resistant to apoptosis and rapidly develop a senescence-associated secretory phenotype (SASP) with hyperactive secretion of cytokines, pro-fibrotic growth factors, and ECM components [3, 4]. Senescent cells are small in number [5] but damaging effects of their pro-inflammatory SASP are widely recognized [6]. Senescent cells have been described as key mediators of tissue fibrosis in part to their ability to promote abnormal and persistent fibroblast activation and ECM remodeling [7].

Our previous work highlighted that radiation-induced senescent MSCs deposit and crosslink matrix proteins altering the architecture and mechanics of the surrounding collagen-rich environment, while secreting cytokines crucial to cell-cell communication [3]. MSCs are stromal cells that are recruited and accumulate in primary tumor tissues due to their natural tropism for inflammatory tissues. Radiation-induced senescence also increased the number and altered the content of secreted exosomes (EXOs) [8]. Senescence-associated EXOs (SA-EXOs) are potent SASP factors that transfer proteins, mRNAs, and miRNAs to mediate disease progression [9]. Previous studies have shown SA-EXOs can activate nearby fibroblasts promoting ECM remodeling through the TGF-β pathway [9, 10]. We also demonstrated the critical role of TGF-β in actin cytoskeletal reorganization to alter cell contractility and ECM remodeling, which are critical steps in the development of fibrosis [11–13]. This local ECM remodeling was associated with increased proliferation and motility of breast cancer cells [3]. We hypothesized that SA-EXOs released by senescent MSCs played a critical paracrine signaling role in inducing these more aggressive breast cancer cell behaviors. These senescent cells are scarce *in vivo*, yet their potency can be transferred to neighboring naïve cells via EXOs and EXO miRNA. High-throughput EXO miRNA profiling from various cell types has been extensively reported [8, 14]. MiRNAs derived from senescent MSCs and subsequent targeting genes, however, need to be further examined. Therefore, in this study we sought out to 1) examine protein and RNA complement differences between pre-senescent and senescent MSCs to determine stand-out pathways that are significantly altered via senescence, 2) use high-content miRNA microarray analysis to examine varying miRNAs and targeted pathways between pre-senescent and senescent EXO populations, and 3) demonstrate how paracrine signaling of SA-EXOs impose potent phenotypes in neighboring cells.

Using integrative pathway enrichment analysis of multiple omics data sets, we demonstrate that radiation-induced senescence enriches for genes associated with cell cycle modifications, cell-matrix interactions, TGF-β signaling, and vesicle transport in MSCs. Genes associated with clathrin- and caveolae-mediated pathways that are critical in EXO biogenesis were significantly upregulated and we found that senescent cells secreted more EXOs than control cells. SA-EXOs were also larger compared to control MSCs and cells treated with TGF-β inhibitors. Next, we used microarrays to analyze the miRNA content of secreted EXOs. MiRNA content analysis of secreted EXOs further revealed that radiation-induced senescence uniquely altered miRNA profiles. In fact, several miRNAs directly targeted TGF-β or downstream genes. To examine bystander effects of radiation-induced senescence, we further treated normal MSCs (control MSCs) with SA-EXOs. This EXO treatment modulated genes related to the TGF-β pathway and elevated both alpha smooth muscle actin (protein increased in senescent, activated cells) and Ki-67 (proliferative marker) compared to untreated MSCs. We revealed SA-EXOs possess unique miRNA content that can influence myofibroblast phenotypes via TGF-β pathway activation. This highlights that SA-EXOs are potent SASP factors that play a critical role in the development of cancer-related fibrosis.

## RESULTS

Senescent MSCs were derived from pre-senescent MSCs that were irradiated with gamma-irradiation. Pre-senescent MSCs were not used beyond passage three. The development of irradiated-induced senescent cell populations was validated and confirmed using B-galactosidase staining and through qRT-PCR probing for senescence-associated markers. Senescent cell populations indicated greater than 80% B-galactosidase positive expression and significant increases in senescence-associated markers.

### Transcriptome pathway regulation of senescent vs. pre-senescent MSCs

**Figure 1:**
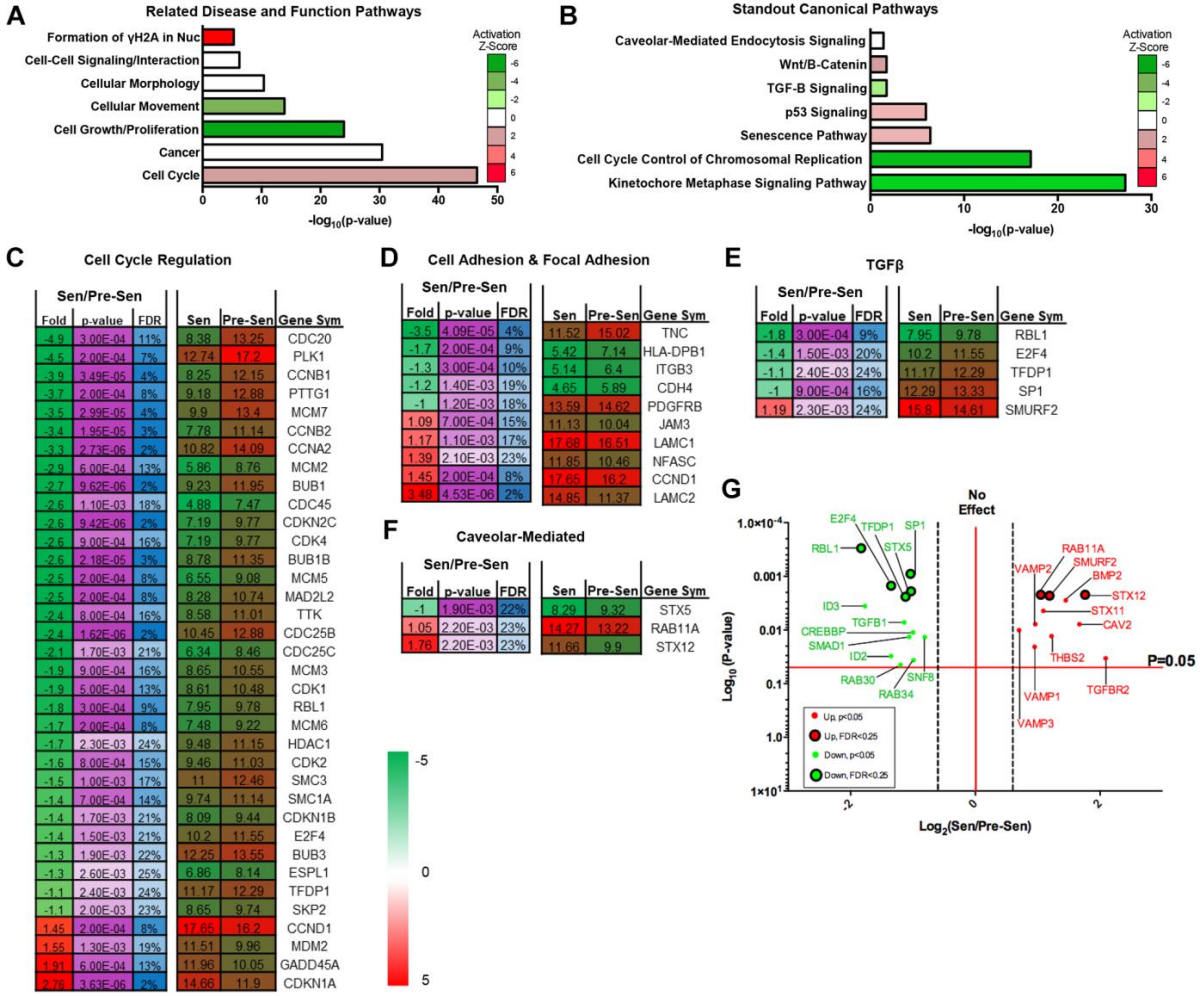
**Senescent MSC transcriptome regulates cell cycle, TGF-β, and vesicle-mediated pathways.** Pre- and senescent MSC transcriptome pathway analysis was performed using Ingenuity Pathway Analysis (IPA). Pathways that were significantly regulated were reported using the (**A**) ‘Related Disease and Function Pathways’ and (**B**) ‘Standout Canonical Pathways’. Pathways that were reported in (**A**) and (**B**) have important overlapping implications. Activation z-scores showed positive enrichment in pathways such as formation of γH2AX in nucleus and cell cycle. These pathways can directly alter the development of senescence phenotype, cell cycle control of chromosomal replication, and kinetochore metaphase signaling. Physical characteristics of cellular movement, growth, and proliferation were downregulated and can affect Wnt/β-Catenin canonical pathway. Cell signaling, morphology, and cancer pathways can correlate to canonical pathways of caveolar-mediated endocytosis, TGF-β, and p53 signaling. List of significantly regulated genes that play important roles in pathways of interest: (**C**) cell cycle regulation, (**D**) cell adhesion and focal adhesion, (**E**) TGF-β, and (**F**) caveolar-mediated. Senescent expression values were normalized to pre-senescent values. Fold changes are reported as log2 differences and genes were limited to those that were FDR < 25%, and *p* < 0.05. More comprehensive gene list for TGF-β and caveolar-mediated pathways (*p* < 0.05) are highlighted in the volcano plots (**G**). Green represents genes that were downregulated and red represents genes that were upregulated. Significant transcripts (*p* < 0.05) with FDR < 0.25 are represented by larger circles with black outlines, whereas significant transcripts (*p* < 0.05) that reported FDR > 0.25 are represented by smaller circles. Several of these genes include TGF-βR2, BMP2, and VAMP isoforms. Pre- and senescent MSC samples were processed (*n* = 4) and differentially regulated pathways were considered significant if *p* < 0.05.

Pre- and senescent MSC mass spectrometry-based transcriptomics was performed (*n* = 3) and processed at Brown University’s Genomics Facility. To determine core regulated pathways between samples, we performed pathway and network analyses using Ingenuity Pathway Analysis (IPA) software. IPA network analysis showed significantly regulated RNA pathways that were involved in mechanosensitive (morphology, movement, and proliferation), cell-cell signaling, and cell cycle pathways (Figure 1A). Z-scores for pathways of interest indicated enhanced activation of molecules regulating γH2Ax formation and cell cycle. Gamma-irradiation used to induce MSC senescence can promote DNA damage and subsequently activate cell cycle arrest genes [15]. We have previously reported increased cell cycle-associated gene regulation in senescent MSCs compared to pre-senescent MSC expression [3]. Conversely, activation Z-scores were downregulated for RNAs associated to cell migration, growth, and proliferation in senescent MSCs. Previous studies from our lab corroborate these transcriptome activation profiles. We highlighted that senescent MSCs reduced velocities (random and directional) and cell proliferation using single-cell migration studies and BrdU staining, respectively [3]. Pathways that were also significantly regulated include morphology, cancer, and cell-cell signaling. Physical characterization studies from our lab have additionally supported these modulated pathways. We assessed that senescent MSCs exhibited stark morphological and area differences compared to pre-senescent MSCs.

Coculture study results between MSCs and breast cancer cells also suggested that senescent MSCs secreted inflammatory factors that induce pro-oncogenic breast cancer phenotypes [3]. These cells could potentially use caveolar-mediated processes as a mechanism for cell-cell communication. Related disease and function pathway analysis was further supported through canonical pathways (Figure 1B). Corresponding cell cycle related pathways (chromosomal replication and kinetochore metaphase signaling), cell growth and proliferation (Wnt/B-Catenin and TGF-β), and cell-cell signaling (caveolar-mediated endocytosis) were significantly regulated. Of these pathways of interest, the caveolar-mediated endocytosis signaling pathway indicated an activation z-score of zero. However, further downstream content analysis and protein expression data indicated that genes related to this pathway were significantly regulated between pre- and senescent MSCs. This supported the importance of examining this pathway regarding its role in secreting important SASP factors. Transcriptome pathway analysis underscored that senescence could alter important MSC pathways that are responsible for dynamic physical and molecular phenotypes and the secretion of SASP factors.

### Senescent MSCs regulate TGF-β and vesicle trafficking gene pathways

Recent work along with our transcriptome profiles showed that TGF-β signaling is dysregulated in senescent MSCs [16, 17]. Studies have also demonstrated that senescent cell-secreted vesicles can enhance aggressive phenotypes during cell-cell interaction via TGF-β signaling pathways [18–20]. Therefore, we further examined significantly altered genes for each of the related transcriptome pathways (Figure 1C–1F). We listed genes associated to cell cycle and mechanosensitive regulation, such as cell and focal adhesion, as these pathways were highly altered. RNAs associated to fold changes between senescent vs. pre-senescent genes were reported as log2 fold differences and were limited to *p*-values < 0.05 and FDR < 25%. A more comprehensive table of genes without FDR cutoff were also included (Figure 1G and Supplementary Figure 1).

We found that SMURF2 was enriched for genes that were involved in the TGF-β pathway (Figure 1E). SMURF2 has been shown to promote senescence and activate cell cycle arrest [21, 22]. SMURF2 gene expression in senescent MSCs could contribute to the activated pathways we observed in Figure 1A. The 11 combined caveolar-mediated associated genes uniformly play important roles in EXO synthesis (Figure 1F, Supplementary Figure 1). Rab11A and STX12 were significantly upregulated in senescent MSCs (Figure 1F). Cells rely on a Rab11A-dependent trafficking pathway to release EXOs [23]. STX12 has been reported as a ubiquitous gene involved in EXO biogenesis and endosomal pathway and trafficking [24, 25]. Other enhanced caveolar-mediated RNAs included LAMP, VAMP, and genes that make up the ESCRT complexes (Supplementary Figure 1) [26]. We subsequently examined the expression of several hallmark genes associated to EXO biogenesis and secretion using qRT-PCR. Gene expression increased in senescent MSCs compared to pre-senescent MSCs (Supplementary Figure 1).

### Integrative omics show senescent MSCs regulate EXO exchange

**Figure 2:**
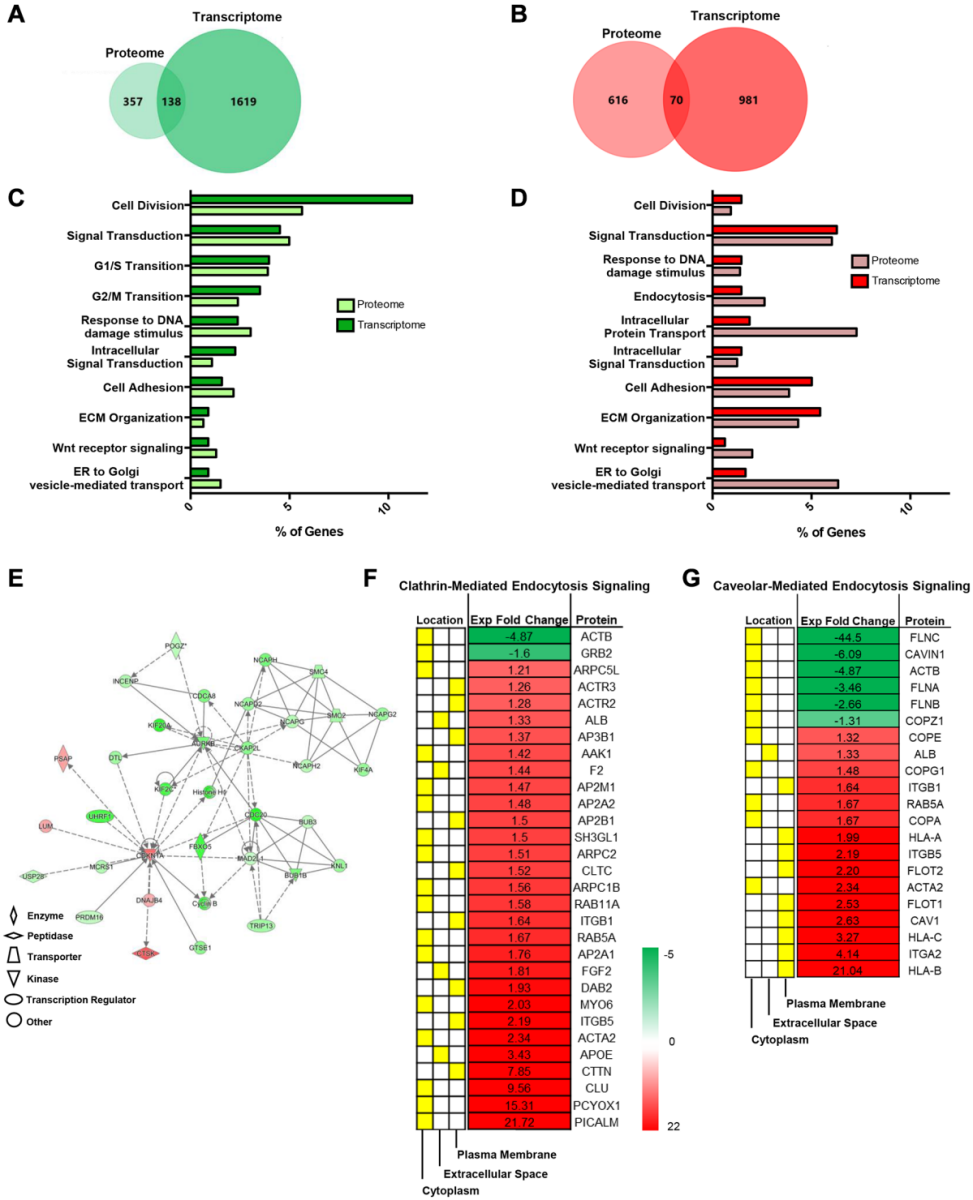
**Integrative omics analysis shows that senescent MSCs regulate endosomal pathways.** Integrative Analysis (proteome and transcriptome) using Functional Enrichment (FunRich) GO Analysis and IPA showed unique and overlapping genes and pathways between pre- and senescent MSCs. Omics analysis showed that 138 overlapping genes were down regulated (green) and 70 overlapping genes were positively enriched (red) (**A**, **B**). The percentage of proteome (light) and transcriptome (dark) genes that are involved in shared biological pathways are listed. These pathways include processes related to cell cycle, extracellular matrix interactions, and vesicle transport (**C**, **D**). Network analysis of genes associated to cell cycle, assembly, organization, DNA replication, cell-cell signaling and interaction, and protein synthesis. Green represents downregulated, red represents upregulated expression, solid lines indicate direct interaction, and dotted lines indicate indirect interaction (**E**). Significant proteins associated to cell-cell signaling, such as endocytic processes (clathrin and caveolin), and respective localized areas (cytoplasm, extracellular space, and plasma membrane) were further reported (**F**, **G**). Senescent MSC expression was normalized to pre-senescent expression. Proteins were down- and upregulated; however, more proteins were enriched in senescent MSCs. Pre- and senescent MSC samples were processed (*n* = 4) and proteins were considered significant if *p* < 0.05.

Because we examined various transcriptome genes that were involved in vesicle trafficking, we further corroborated this trend using integrative omics analysis using Functional Enrichment (FunRich) GO analysis and IPA software. Integrative omics analysis indicated overlapping biological processes for down (green)- and upregulated (red) genes that were of interest (Figure 2A, 2B). Specifically, Venn diagrams show that 138 downregulated and 70 enriched genes overlapped between proteome and transcriptome expression profiles (Figure 2A, 2B).

We previously revealed that radiation-induced senescent MSCs amplified various proteome collagen isoforms related to matrix synthesis and turnover [3]. Senescent cell accumulation develops senescence-associated secretory phenotype (SASP) that is associated to the increased production of inflammatory cytokines, matrix degradation enzymes, and EXOs. We have shown that senescent MSCs elevate the secretion of various interleukins and matrix metalloproteinases. Integrative analysis not only corroborated this but has also demonstrated increased involvement of proteome processes related to intracellular protein and vesicle transport (Figure 2C, 2D). Network of specific genes related to biological processes are further highlighted (Figure 2E).

Studies have shown that clathrin- and caveolar-mediated signaling pathways are crucial in both transport processes [27]. Differentially regulated proteins in both pathways (*p* < 0.05, fold differences) were reported (Figure 2F, 2G). In fact, studies have shown that numerous cell types internalize EXOs using clathrin- and caveolar-mediated endocytosis and proteins involved in these pathways are also important in EXO synthesis [28]. Several proteins listed in Figure 2F, 2G were altered in irradiation-induced SASP and SASP EXOs in various cell lines. These proteins include CAV1, flotillin, and RAB isoforms. Senescence further alters EXO proteome signatures by the enhanced expression of cell adhesion, RAS, and cell junction assembly proteins. This unique proteome profile makes SA-EXOs potent SASP factors/mediators during cell-cell communication.

### Senescence induces unique EXO miRNA profiles

**Figure 3:**
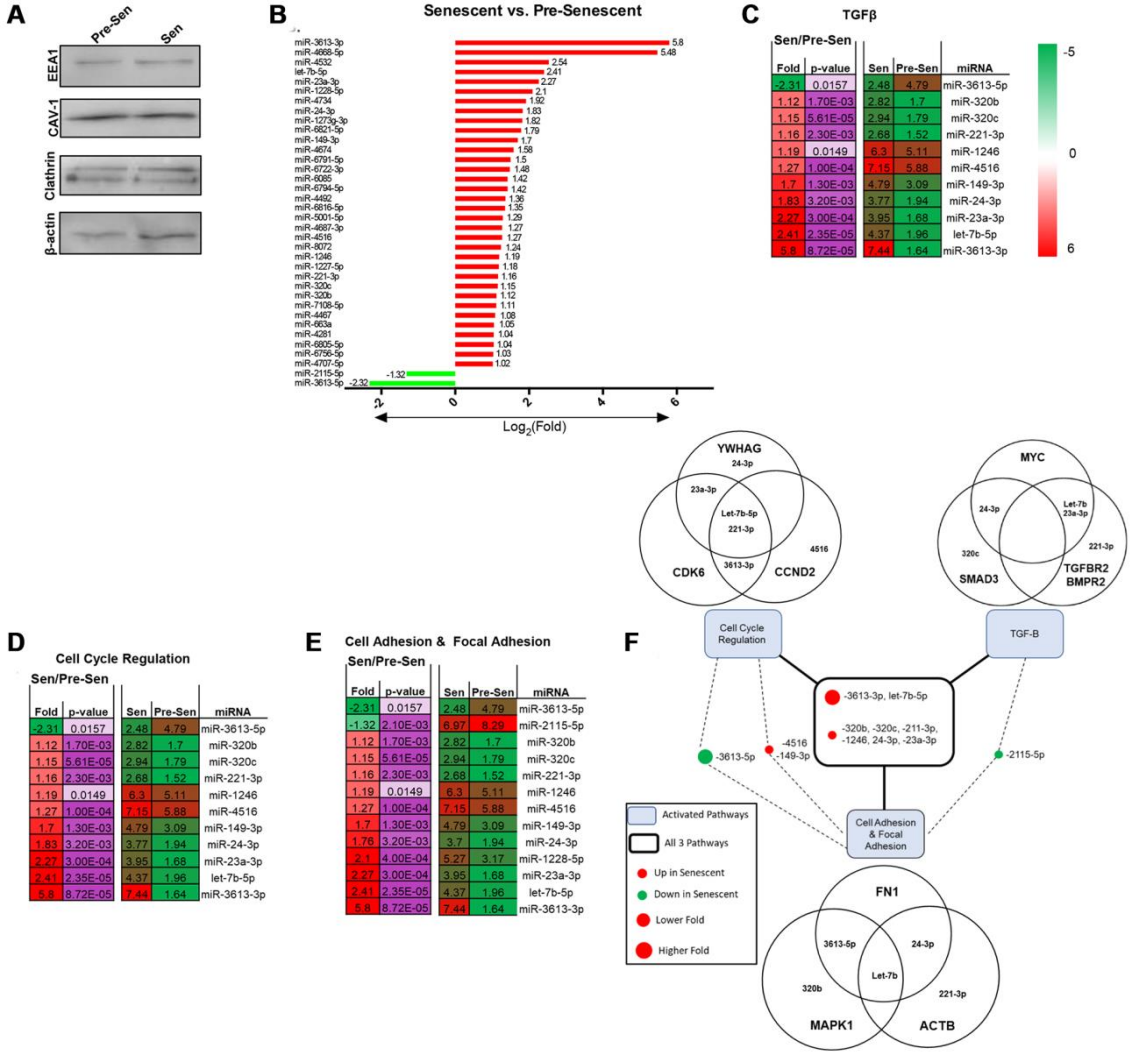
**Senescent MSCs secrete unique EXO miRNA profile.** MiRNA content from pre- and senescent MSC secreted EXOs were characterized and examined using immunoblots and microarrays. Pre-senescent and senescent harvested EXOs showed positive expression of EXO surface markers (EEA1, CAV-1, clathrin, β-actin) using immunoblots (**A**). Many miRNAs that were significantly regulated were positively enriched in senescent MSC EXOs compared to pre-senescent MSC EXOs (senescent vs. pre-senescent) (**B**). Microarray analysis showed overlapping miRNAs were involved in similar transcriptome pathways. Significant miRNAs that were involved in TGF-β (**C**), cell cycle regulation (**D**), and cell adhesion and focal adhesion (**E**) pathways are listed. DIANA miRPath analysis further showed miRNAs that regulated genes reported in transcriptome analysis (**F**). Venn diagrams indicate that several miRNAs regulate and target multiple genes in different pathways. miRNA microarray samples were processed (*n* = 3) and miRNAs were considered significant if *p* < 0.05 and fold changes are reported as log2 differences.

miRNAs are conserved non-coding RNAs that can modulate post-translational gene expression via EXO exchange. EXO miRNA content therefore was examined using microarrays to further assess the role of senescence. Immunoblots probing for EXO positive markers –EEA1, CAV1, Clathrin, and β-actin—were used to validate EXO populations prior to array analysis (Figure 3A) [29]. 43 total miRNAs were differentially regulated; 39 miRNAs were up- and 4 were downregulated. Reported miRNAs were condensed to those that had 2-fold expression level differences and *p* < 0.05. Thus, 36 EXO miRNAs were significantly regulated between senescent vs. pre-senescent MSCs (Figure 3B). 34 miRNAs were up- and 2 miRNAs were down-regulated. miRNA pathway analysis was performed using DIANA tool miRPath. Many overlapping miRNAs were involved in pathways (cell cycle regulation, cell and focal adhesion, and TGF-β) that were significant in transcriptome analysis (Figure 3C–3E).

Pathway analysis also highlighted and correlated miRNAs to genes that were regulated in Figures 1 and 2. In particular, miRNA 3613-3p and let-7b-5p were involved in all three pathways and highly upregulated with log2 fold changes of 5.8 and 2.41, respectively (Figure 3F). Both miRNAs positively modulate cell cycle genes CDK6 and CCND2. CCND2 regulates CDK6, a kinase that controls G1/S transition [30]. Further, miRNA-221-3p and let-7b-5p were shown to regulate ACTB. Cellular senescence can regulate cytoskeletal proteins, such as actin. We have previously shown that senescent MSCs reorganize filamentous actin structures, where actin fibers were longer and polarized [3]. Senescence uniquely modulating various actin isoforms can contribute to biophysical changes highlighted in transcriptome pathways, such as morphology and migration. miRNA-23a-3p was also involved in all pathways and regulates TGF-βR2 expression. TGF-βR2 was enhanced in senescent MSCs (Figure 1G). Studies have shown miRNA-23a promotes TGF-β expression and regulates aggressive phenotypes [31]. Taken together, these results indicate that radiation-induced senescence propagates unique EXO miRNA packaging which directly modulate genes important in TGF-β and mechanosensitive pathways.

### TGF-β pathway alters EXO miRNA content in senescent MSCs

**Figure 4:**
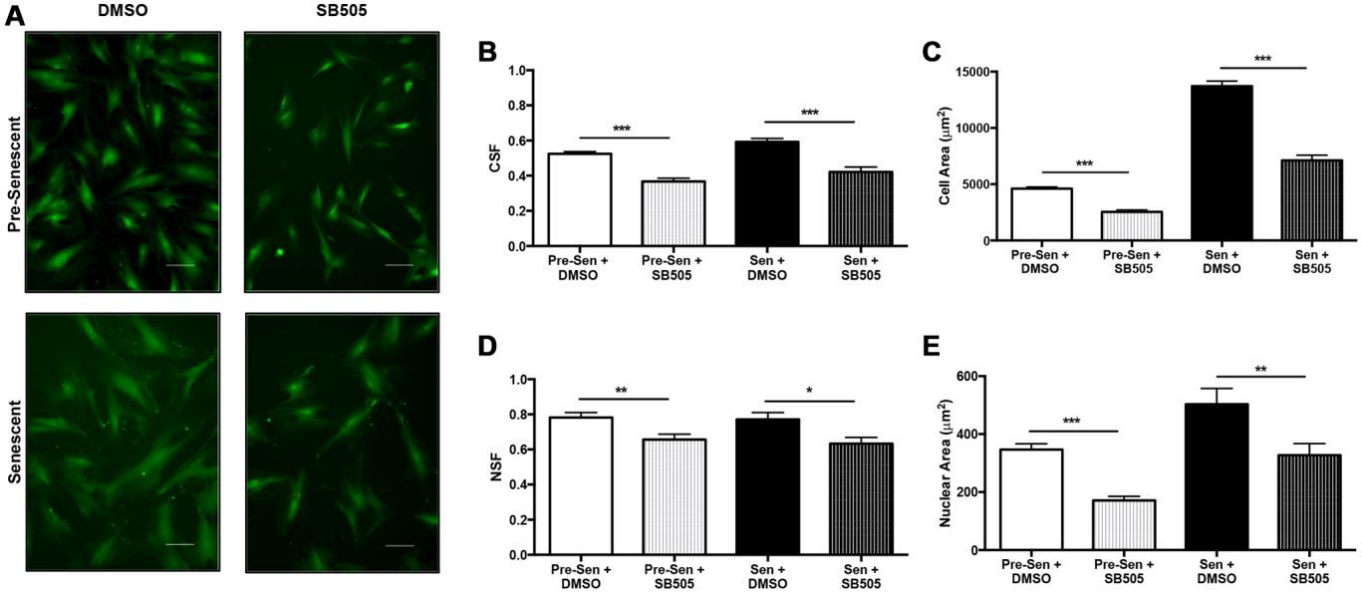
**TGF-β affects MSC physical phenotype.** Pre- and senescent MSCs were treated with 10 μM SB505-124 (SB505) for 10 days every 72 hours. MSCs that were treated with DMSO served as the control groups. MSCs were stained with Calcein-AM and 10X representative images were taken after 10 days (**A**). ImageJ shape parameter tools were used to measure cell shape factor (CSF) (**B**), area (**C**), nuclear shape factor (NSF) (**D**), and nuclear area (**E**) for each cell with ~500 cells per condition. Treatment with SB505 elongated pre- and senescent cell and nuclear morphologies. This treatment also decreased cell and nuclear areas. Shape factor value of 1 represents a circle and 0 represents a line. Scale Bar: 100 μm. ^*^p < 0.05, ^**^p < 0.005, ^***^p < 0.001.

TBF-β is a ubiquitous cytokine in the development of senescence and its downstream pathway genes mediate important physical and molecular phenotypes. Array analysis further indicated that various EXO miRNAs were involved in this pathway. We sought to investigate the difference in EXO miRNA expression profiles when inhibiting TGF-β in pre and senescent MSCs. Briefly, pre and senescent MSCs were treated with SB505-124, a small-molecule inhibitor of TGF-βR1. SB505 treatment altered cell and nuclear phenotypes compared to untreated cells (Figure 4A). ImageJ cell shape factor (CSF) and nuclear shape factor (NSF) were used to measure morphologies where the values of 1 and 0 represent a circle and line, respectively. CSF measurements showed that pre and senescent MSCs elongated and cell areas decreased with SB505 treatment compared to cells treated with DMSO (control) (Figure 4B, 4C). Similarly, NSF and nuclear area measurements decreased with SB505 treatment (Figure 4D, 4E).

**Figure 5:**
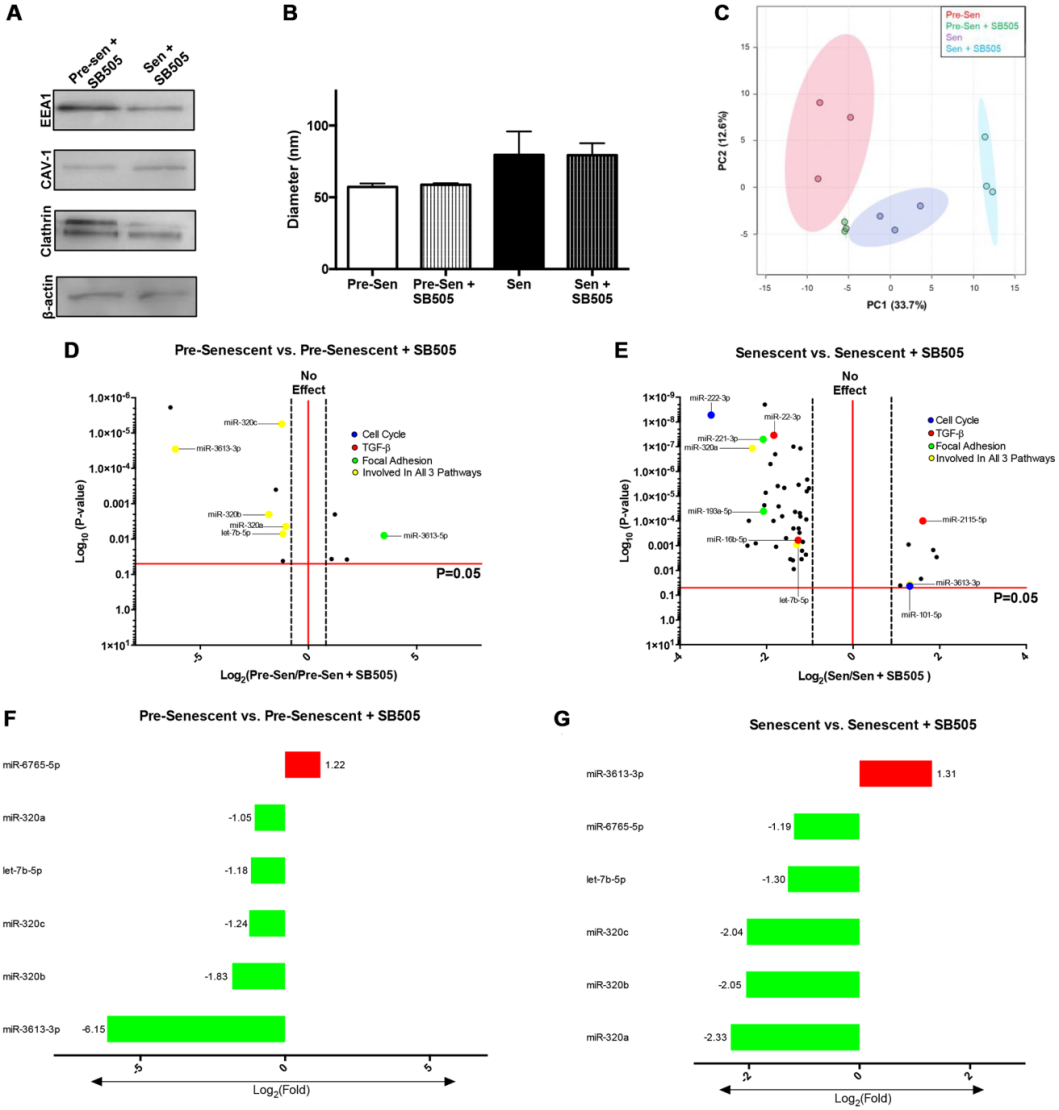
**TGF-β modulates EXO miRNA profiles in pre- and senescent MSCs.** EXO surface markers and miRNA content from pre- and senescent MSCs treated with SB505 were analyzed using immunoblots and microarrays. EXO populations were validated using immunoblots probing for well-known surface markers (**A**). Diameters were measured using dynamic light scattering methods and indicated similar diameters between untreated and SB505-treated EXOs from pre- and senescent MSCs; however, senescent MSC EXOs were larger than EXOs from pre-senescent cells (**B**). Principal component analysis (PCA) for pre- and senescent MSC EXOs treated with and without SB505 were plotted (pre-sen: red, pre-sen + SB505: green, sen: purple, sen + SB505: blue). Each point indicates a biological sample (*n* = 3) and the first two principal components explain 46.3% of miRNA variance (**C**). DIANA miRPath analysis showed that many of the significantly regulated miRNAs were involved in pathways of interest: cell cycle (blue), TGF-β (red), focal adhesion (green), and regulated in all 3 pathways (yellow) (**D**, **E**). 6 miRNAs overlapped between pre-senescent vs. pre-senescent + SB505 (**F**) and senescent vs. senescent + SB505 (**G**). In particular, expression levels between miRNAs-6765-5p and -3613-3p were inversely related between conditions. miRNA microarray samples were processed (*n* = 3) and miRNAs were considered significant if *p* < 0.05 and fold changes are reported as log2 differences.

Secreted EXOs were harvested from SB505-treated and untreated pre and senescent MSCs. Immunoblots probing for EXO positive markers—EEA1, CAV-1, clathrin, and β-actin—were used to validate EXO populations (Figure 5A). Physical characteristics, such as EXO diameters, between treated and untreated MSCs were comparable (pre-sen: 57.26 nm ± 13.18; pre-sen + SB: 58.665 ± 10.24; sen: 79.54 nm ± 18.62; sen + SB: 79.29 ± 11.37). However, we found that senescent MSCs that were treated or untreated with SB505 secreted larger EXOs (Figure 5B). Principal component analysis (PCA) of pre-senescent EXOs and SA-EXOs treated with or without SB505 suggest distinct differences in miRNA content (Figure 5C). PCA also indicate reduced heterogeneity in EXO miRNA content treated with SB505.

miRNA pathway analysis using DIANA tools showed that EXO miRNAs overlapped and were significantly involved in pathways that were examined in transcriptome analysis (Figure 5D, 5E). Using same cutoff conditions, significantly regulated miRNAs for each condition were examined and were largely down-regulated EXOs harvested from pre-senescent MSCs ± SB505 had 12 differentially regulated miRNAs—four that were up- and eight that were down-regulated. EXOs isolated from senescent MSCs ± SB505 had 51 differentially regulated miRNAs—eight that were up- and 43 that were down-regulated (Supplementary Figure 2). miRNAs-320a-c, 6765-5p, 3613-3p, and let-7b-5p were shared between conditions (Figure 5F, 5G). miRNAs-320a-c fold changes were attenuated in senescent compared to pre-senescent conditions. This miRNA cohort has been reported to directly target various genes in the TGF-β pathways and modulate tumorigenesis and pro-fibrotic changes [32]. Previous studies have shown that miRNA-3613-3p expression was elevated in EXOs isolated from activated cancer fibroblasts and was responsible for initiating metastatic tumor cell behavior [33]. Let-7b-5p is a widely examined miRNA that plays significant roles in various pathways and disease mechanisms. Interestingly, miRNAs- 6765-5p and -3613-3p were inversely related between pre-senescent MSCs ± SB505 EXOs and senescent MSCs ± SB505 EXOs. miRNA microarray analysis showed that blocking TGF-β pathway via inhibiting TGF-βR1 directly affects EXO miRNA content in pre and senescent MSCs.

### Senescent MSC EXO exchange activates myofibroblast phenotype

**Figure 6:**
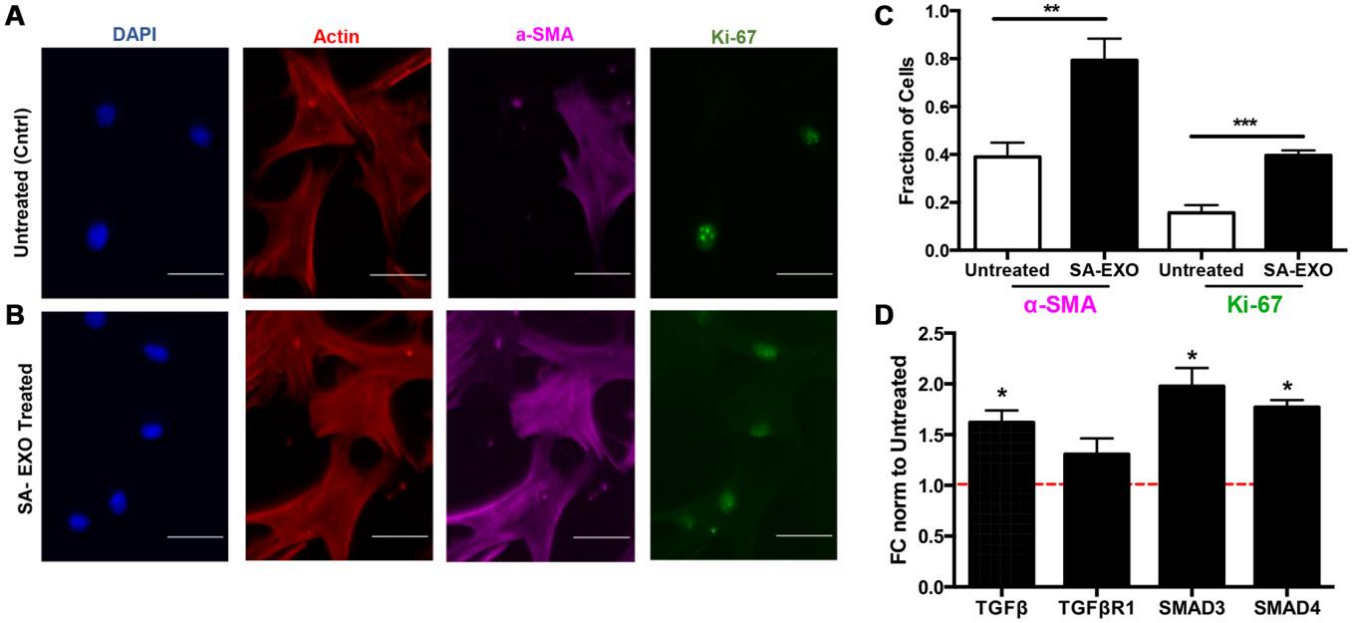
**Senescent EXO exchange promotes activated MSC phenotype.** Pre-senescent MSCs were treated with or without senescent MSC EXOs (SA-EXOs) and stained for dapi (blue), filamentous actin (red), a-SMA (cyan), and Ki-67 (green) (**A**, **B**). EXO treatment elevated a-SMA and Ki-67 expression compared to untreated MSCs (**C**) and further modulated TGF-β related gene profiles (**D**). Gene expressions were normalized to β-actin and to untreated MSCs (control and represented by dashed red line). Scale Bar: 100 μm. ^*^p < 0.05, ^**^p < 0.005, ^***^p < 0.001.

Senescence-derived EXOs and cohorts of miRNAs have been shown to activate invasive phenotypes during cell-cell communication. Thus, we sought to examine if SA-EXO treatment led to pre-senescent MSCs exhibiting myofibroblast-associated characteristics. Briefly, pre-senescent MSCs were treated with SA-EXOs and stained for DAPI, filamentous actin, a-SMA, and Ki-67 (Figure 6A). SA-EXO treatment amplified ~2-fold of both a-SMA and Ki-67 expression compared to control, untreated MSCs (Figure 6B, 6C). Increased expressions indicate that SA-EXOs can play important roles in triggering fibroblast differentiation into potent, activated myofibroblasts [33–36].

Because our integrated omics analysis and miRNA microarray data highlighted that TGF-β genes were highly regulated, we subsequently examined genes critical in this pathway. qRT-PCR results revealed that SA-EXO treatment significantly increased TGF-β, SMAD3, and SMAD4 expressions in pre-senescent MSCs (Figure 6D). Studies have shown that canonical and non-canonical TGF-β pathways regulate stem cell differentiation [37]. Therefore, our results suggest that SA-EXOs can increase a-SMA and Ki67 expressions and TGF-β genes, which are indicative of an activated and invasive MSC.

## DISCUSSION

Bone marrow-derived MSCs can naturally undergo senescence. These cells can develop and secrete SASP that is linked to hyperactive secretion of cytokines, fibrotic growth factors, and ECM components [38]. The accumulation of these factors alters the pathophysiology of cancer progression and may further accelerate the aging process. Senescent cells are scarce, but their SASP has powerful effects on *in vivo* tissue environments [39].

Senescent vs. pre-senescent MSC integrative omics analysis showed that biological processes included cell cycle regulation, response to DNA damage stimulus, cell-matrix interactions, and vesicle-mediated transport were highly dysregulated. Integrative proteomic and transcriptomic analysis of genes involved in these pathways were simultaneously downregulated and enriched. These observations could be partly attributed to the heterogeneous nature of MSCs undergoing senescence [40]. We have previously reported, through proteomics and high-throughput content analysis, that senescent MSCs increased expression of ECM proteins and released a more potent secretory phenotype. Together, these factors influenced the development of invasive neighboring cell phenotypes.

Previous proteomics data showed that thousands of proteins were significantly regulated between pre- and senescent MSCs. However, our recent integrative analysis reported that approximately only hundreds (~10-fold reduction) of these genes were differentially modulated at both the RNA and protein levels. Integrative omics analysis is necessary for understanding complex biological processes, such as senescence, and the pathways that are involved in senescence associated secretory phenotype development. The specific analytical approach reflects the dual protein and RNA interactions and subsequently reveals the functional understanding of differentially regulated genes; this can help target the specific gene and protein candidates that are responsible for propagating or mitigating biological pathways involved in senescence-associated disease.

Our integrated omics analysis showed similar trends in pathway regulation. Many of the pathways that were important in regulating senescence overlapped, but the number of regulated genes varied between proteome and transcriptome profiles—this clearly indicated differences in transcriptional and post-transcriptional regulation. For instance, more proteome genes were upregulated in vesicle mediated transport in senescent MSCs, whereas more transcriptome genes were downregulated in cell division pathways. Integrative omics analysis additionally highlighted that radiation-induced senescence modulates mechanisms critical to multiple signal transduction processes, such as intracellular protein transport and Wnt-receptor signaling activity.

EXOs have emerged as potent SASP factors that play pivotal roles in cellular crosstalk as studies have shown that their miRNAs can alter molecular and physical phenotypes in recipient cells [41]. Transcriptome analysis revealed that pathways involved in “cell-cell signaling interactions” and “caveolar-mediated endocytosis signaling” were significantly altered. Senescence was also characterized by the elevated production of EXOs and EXOs that were also larger in size. Senescent MSCs enriched protein expressions, such as CAV2, STX, and VAMP isoforms, that are crucial in exosome biogenesis and secretion. Microarray analysis showed that many of the miRNAs (senescent vs. pre-senescent) were upregulated in EXOs isolated from senescent MSCs. In fact, these miRNAs modulated protein expression that were involved in highlighted integrative pathways (TGF-β, cell cycle regulation, and cell and focal adhesion).

Standout SA-EXO miRNAs that were positively activated included miRNA-3613-3p and 4668-5p (log2 FC 5.8 and 5.48, respectively). Previous studies indicated miRNA-3613-3p play important roles in disease progression [33, 42, 43]. Upregulation of miR-313-3p has been reported in various carcinomas, such as lung, colon, and breast. 3613-3p can serve as a predictive biomarker to chemotherapeutic response for women with breast cancer, where overexpression correlates to a favorable prognosis [42]. This miRNA overexpression can attenuate the proliferation of breast cancer cells by G0/G1 cell cycle arrest and promote cellular senescence. More importantly, it was revealed that SMAD2 and EZH2 were target genes of 3613-3p. SMAD2 is a critical transcription factor in the TGF-β pathway; this indicates that 3613-3p can modulate genes downstream of SMAD2 [43]. EZH2 is a transcription factor in the E2F family that is important in cell cycle regulation [44]. SMAD and E2F isoforms were enriched our integrative analysis. Similarly, 3613-3p was upregulated in EXOs harvested from cancer-associated fibroblasts that were pre-treated with TGF-β and in breast cancer fibroblasts that were cocultured with breast cancer cells [33]. Liu et al., however, found that CAF miRNA-3613-3p-inhibited EXOs reduced breast cancer cell proliferation and metastasis. This was marked by the reduced migration and invasion profiles upon EXO treatment. These studies indicated that 3613-3p could function as a tumor suppressor and act as a therapeutic target in breast cancer metastasis [33]. Our previous findings and our microarray analysis further corroborate these findings as 3613-3p is elevated in senescent MSCs and these senescent MSCs activated more invasive breast cancer cell phenotypes in co-culture. miR-3613-3p could have played a role in activating invasive breast cancer cell phenotypes in our spheroid model during MSC-breast cancer cell crosstalk. miRNA-4668-5p was positively regulated in senescent MSC exosomes. This miRNA has emerged as a novel biomarker as it is consistently elevated in the serum of pancreatic cancer, hepatocellular carcinoma, and liposarcoma patients [45–47]. It is also predicted to regulate the TGF-β pathway via targets, such as TGF-βR1 and SMAD isoforms.

Conversely, miRNAs-2115-5p and 3613-5p were significantly downregulated in senescent MSC EXOs. Very little has been reported regarding the role of 2115-5p. However, reduced expression of miRNA-3613-5p was consistently found in pancreatic cancer patients and correlated to poor disease prognosis [48]. This study found that miR-3613-5p regulated CDK6 expression and that downregulating 3613-5p in pancreatic cancer cells led to faster scratch wound closure and accelerated transwell invasion capacities [48]. Senescent vs. pre-senescent MSC integrative analysis showed CDK isoform enrichment. Combining previous studies and the microarray analysis, we show that significantly up- and downregulated senescent miRNAs can target genes that elicit invasive phenotypes in recipient cells during EXO exchange. Our microarray data revealed a novel, differentially regulated miRNA repertoire that can uniquely target genes important for mechanosensitive and TGF-β pathways. Genes regulating these pathways have been shown many times to play important roles in tumor and fibrotic disease progression. The prevalence of tumor and fibrotic disease is age-dependent ad aging is associated with increased numbers of senescent cells. Thus, it is important to examine the unique roles these EXO-encapsulated miRNAs play in senescence development.

Senescence further correlates with increased TGF-β signaling. Proteomics and transcriptomic analysis indicate that activated genes are involved in this pathway. TGF-β is a pleiotropic cytokine that plays a critical role in reorganizing cytoskeletal proteins, acquiring cellular senescence, and modulating expression of downstream canonical and noncanonical target genes. Many of the regulated miRNAs we reported targeted TGF-β or critical downstream pathway genes. Inhibiting autocrine and paracrine TGF-β signaling via SB505 elongated senescent and pre-senescent MSCs and nuclear morphologies. SB505 also led to increased heterogeneity in pre and senescent MSC populations (Supplementary Figure 3). Studies have shown that TGF-β induces actin stress fiber assembly and TGF-β inhibition disrupts polymerization [49]. Cytoskeletal proteins, such as cortactin, control vesicular trafficking and fuse late endosomes with the plasma membrane [50]. These proteins promote EXO secretion by stabilizing actin-rich docking sites. TGF-β not only affects cytoskeletal regulation and EXO secretion, but we have revealed that it can also lead to secreting unique EXO miRNA content.

Microarray analysis showed that numerous miRNAs were involved in pathways that were highlighted previously via integrative analysis. Further, microarray SB505 analysis highlighted that a TGF-β inhibitor leads to unique EXO miRNA packaging and secretion in pre and senescent MSCs. Positively regulated miR-3613-3p (senescent vs. pre-senescent) was also enriched after SB505 treatment for senescent MSC EXO analysis. Treatment with SB505 (senescent vs. senescent +SB505) reduced -3613-3p fold change expression compared to senescent vs. pre-senescent. This indicates that TGF-β could play a direct role in -3613-3p expression when acquiring a senescent MSC phenotype. Previous studies have directly linked increased expression of exosome -3613-3p to activating invasive cellular and mechanical properties [33, 51]. Therefore, reduced expression of 3613-3p with SB505 treatment could indicate that these MSCs possess limited activation potential to acquire CAF-like phenotypes. Interestingly, pre-senescent MSC EXOs treated with SB505 exhibited higher levels of 3613-3p than untreated pre-senescent MSC EXOs (control). This shows that TGF-β and its downstream genes play differential roles in pre vs. senescent MSCs EXO synthesis and biogenesis. miR-6765-5p also exhibited inverse relations—positively enriched in pre-senescent MSCs and negatively enriched in senescent MSCs compared to SB505 treatment. Few studies have reported significant expression of this miRNA and its target genes. Therefore, this miRNA potentially serves as a novel senescence biomarker that targets the TGF-β pathway.

**Figure 7:**
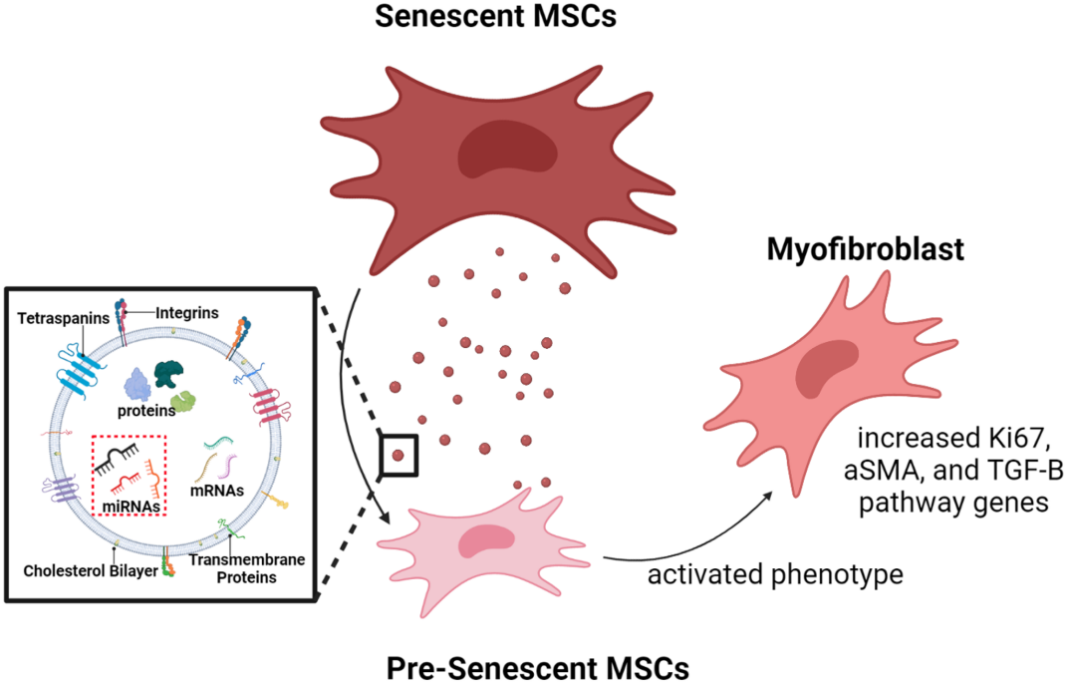
**Graphical illustration.** Radiation-induced senescent MSCs secrete SA-EXOs that encapsulate unique miRNA profiles, compared to normal MSCs (control). These miRNAs target and modulate genes and proteins that are crucial in various mechanosensitive and TGF-β pathways. SA-EXO exchange between normal MSCs and SA-EXOs activated potent phenotypes—these phenotypes included elevated a-SMA, Ki67, and TGF-β mediated gene expressions. This shows that SA-EXOs can serve as potent SASP mediators that activate invasive characteristics in neighboring cells.

Our integrated omics and EXO microarray analyses show that senescent MSCs possess differential transcriptional genes and secrete vesicles that contain unique post-transcriptional cargo. We subsequently demonstrated that these EXO miRNAs can play important roles in cell-cell communication during disease progression. Increases in a-SMA, ki-67, and TGF-β genes indicate that SA-EXOs are potent SASP factors when activating invasive MSC phenotypes [16, 52]. The observed and quantified changes we reported after SA-EXO exchange highlight that these EXOs contain cargo that mediates the TGF-β pathway and amplifies SASP potency (Figure 7).

## MATERIALS AND METHODS

### Mesenchymal stem cell (MSC) cell culture

Female bone marrow-derived MSCs (8001L; Texas A&M Cell Distribution Center) were maintained in Minimum Essential Medium Alpha (Corning), supplemented with 20% Fetal Bovine Serum (FBS) (Atlanta Biologicals), 1% Penicillin-Streptomycin (Corning), 15 mM HEPES (Fisher Chemicals) and 1% L-Glutamine (Corning). Cell culture media was replaced every 72 hours and passage number did not exceed six due to natural senescence drift MSCs exhibited. MSCs were grown at 37°C and 5% CO_2_.

### Irradiation-induced senescence

Unfractionated, single-dose gamma irradiation of 15 Gy was administered to MSCs (Mark I 68A137Cs Irradiator). Senescence was confirmed 10 days post irradiation using β-galactosidase staining and qRT-PCR probing for a panel of senescence markers. These validation methods have been extensively reported by our lab in previous publications [3].

### SB505-124 treatment

Irradiated and pre-senescent MSCs were treated with 10 μM of SB505-124 (SB505) immediately after irradiation for every 72 hours for 10 days.

### miRNA microarray

Total EXO RNA was isolated according to manufacturer’s protocol (Total EXO and RNA and Protein Isolation Kit, Invitrogen). EXOs from MSC populations (pre, senescent, pre + SB505, and senescent + SB505) were resuspended in EXO Resuspension Buffer (Invitrogen). EXO RNAs were extracted using acid-phenol chloroform, washed, and eluted through filter cartridges. 10 μg of EXO miRNA was labeled for Affymetrix array analysis (NuGEN Technologies). Sample array hybridization was performed at Brown University’s Genomics Core Facility.

### Transcriptome sample processing

Pre- and senescent MSCs were prepared in triplicate for gene expression analysis (*n* = 3). Cells were washed in PBS and suspended in PureZOL before storing samples at −80°C. RNA was isolated using standard chloroform extraction methods as per manufacturer's protocol. 100 ng of total RNA was amplified using Affymetrix Sensation Plus FFPE amplification kit following manufacturer’s instructions. Labeled cDNA hybridized to Affymetrix Clariom-D microarrays and visualized at Brown University’s Genomics Core Facility.

### Bioinformatics analysis

#### Proteome and transcriptome

Proteins and RNAs that had *p* < 0.05 were considered significant. Enrichment analysis of significantly regulated genes was done using QIAGEN Ingenuity Pathway Analysis software. IPA software was used to evaluate transcriptome and integrate proteome and transcriptome data between pre- and senescent cell populations. Functions such as ‘Pathway analysis, Canonical pathways, and Overlapping pathways’ were used to identify biological targets and standout pathways. Z-scores were obtained via IPA. Activation z-score serves as a predictive metric if a pathway is either activated or inhibited. This score is determined using the expression of a cohort of molecules downstream of target regulators (upstream). A pathway is considered activated if z ≥ 2 and inhibited if z ≤ −2. Further, a pathway can be considered as “no activity prediction” if there are fewer than four regulated molecules in the desired pathway.

#### miRNA microarray

miRNAs that had *p* < 0.05 were considered significant. miRNAs were uploaded to DIANA miRPath for subsequent pathway analysis.

### Exosome isolation

MSCs (pre-senescent and senescent) media was replaced with EXO-depleted serum media 48 hours prior to exosome collection. EXOs were harvested using standard ultracentrifugation protocols that have been extensively used in previous studies.

### Immunoblotting

EXOs (pre-, senescent, pre-treated with SB505, and senescent treated with SB505) were collected and immediately lysed and heated for five minutes. Samples were separated using sodium dodecyl sulfate polyacrylamide (SDS-PAGE) gels and transferred onto polyvinylidene difluoride (PVDF) membranes (Thermo Fisher). PVDF membranes were proved using primary antibodies—EEA1, CAV-1, Clathrin, β-actin (Cell Signaling Technology). Bands were visualized using Clarity ECL Western Substrate (BioRad). Full blot images are included in Supplement (Supplementary Figure 4).

### Cell and nuclear analysis

MSCs were labeled with 1 μM Calcein-AM and 1 μg/mL of Hoechst (BioLegends) and imaged at 10X magnification. Morphologies and areas (cell body and nucleus) were quantified using an ImageJ shape factor parameter plugin. Shape factor of 1 represents a circle, whereas shape factor of 0 represents a line.

### Quantitative real time-polymerase chain reaction (qRT-PCR)

Total RNA was isolated using Ribozol (BioRad). 1 μg of total RNA was converted to cDNA using an iScript cDNA synthesis kit (BioRad). The cDNA was then used for qRT-PCR to analyze TGF-β pathway related genes. Expression values were normalized to β-actin and untreated MSCs (control). Genes are listed in Supplementary Table 1 and primers were purchased from IDT.

### Immunocytochemistry (actin, a-SMA, and Ki-67)

Pre-senescent MSCs were cultured at 60% confluency on coverslips. Cells were subsequently pre-treated with SA-EXOs. Briefly, pre-treated cells were fixed with 4% paraformaldehyde (Thermo Scientific), permeabilized with 0.5% Triton X-100 (Fisher Bioreagents), and blocked with 5% bovine serum albumin (Alfa Aesar). Cells were then incubated in a-SMA (1:1000) and Ki67 (1:1000) primary antibodies overnight (Cell Signaling Technologies). Cells were then washed and next stained with 1:200 Rhodamine Phalloidin (Invitrogen), 1:500 of Alexa Fluor 488 goat anti-rabbit (secondary antibody for a-SMA) (Invitrogen), and Alexa Fluor 555 goat anti-mouse (secondary antibody for Ki-67) (Invitrogen). Coverslips were finally mounted using Vectashield Mounting Medium with DAPI (SouthernBiotech). 40X resolution images were taken with Nikon Eclipse Ti microscope.

### Statistics for integrative omics analysis and microarray analysis

Pre- and senescent MSC samples (proteome and transcriptome) were processed (4 biological replicates for proteome and 3 biological replicates for transcriptome) at Brown University’s Genomics Core Facility. Senescent MSC gene profiles were normalized to pre-senescent MSCs profiles. Shared genes between senescent vs. pre-senescent MSCs were considered significant if *p* < 0.05. A differentially expressed cutoff of FDR < 0.25 was further applied for genes reported in main figures. MiRNA microarray samples were processed (3 biological replicates) for pre- and senescent MSC EXOs that were treated with or without SB505. Similarly, miRNAs were considered significant if *p* < 0.05. SA-EXO miRNAs were compared to pre-senescent MSC miRNA EXOs; SA-EXO miRNAs were compared to SA-EXO +SB505 miRNAs; pre-senescent EXO miRNAs were compared to pre-senescent EXO + SB505 miRNAs.

### Statistics

Data in manuscript are reported as mean ± standard error of the mean. All experiments were performed at least three times with at least three technical replicates per experiment. Student’s *t*-tests were calculated to determine significance (*p* < 0.05). ^*^p < 0.05, ^**^p < 0.005, ^***^p < 0.001. EXO treated groups or SB505 treated groups were compared to untreated or respective controls.

## Supplementary Materials

Supplementary Figures

Supplementary Table 1
