# Supplementary material for: Senescence-associated exosomes transfer miRNA-induced fibrosis to neighboring cells
Source: Aging (Albany NY). 2023 Feb 23;15(5):1237–56. doi: 10.18632/aging.204539 (PMC10042705; doi:10.18632/aging.204539)
Supplement: Supplementary Figures [file aging-15-204539-s001.pdf]

SUPPLEMENTARY FIGURES

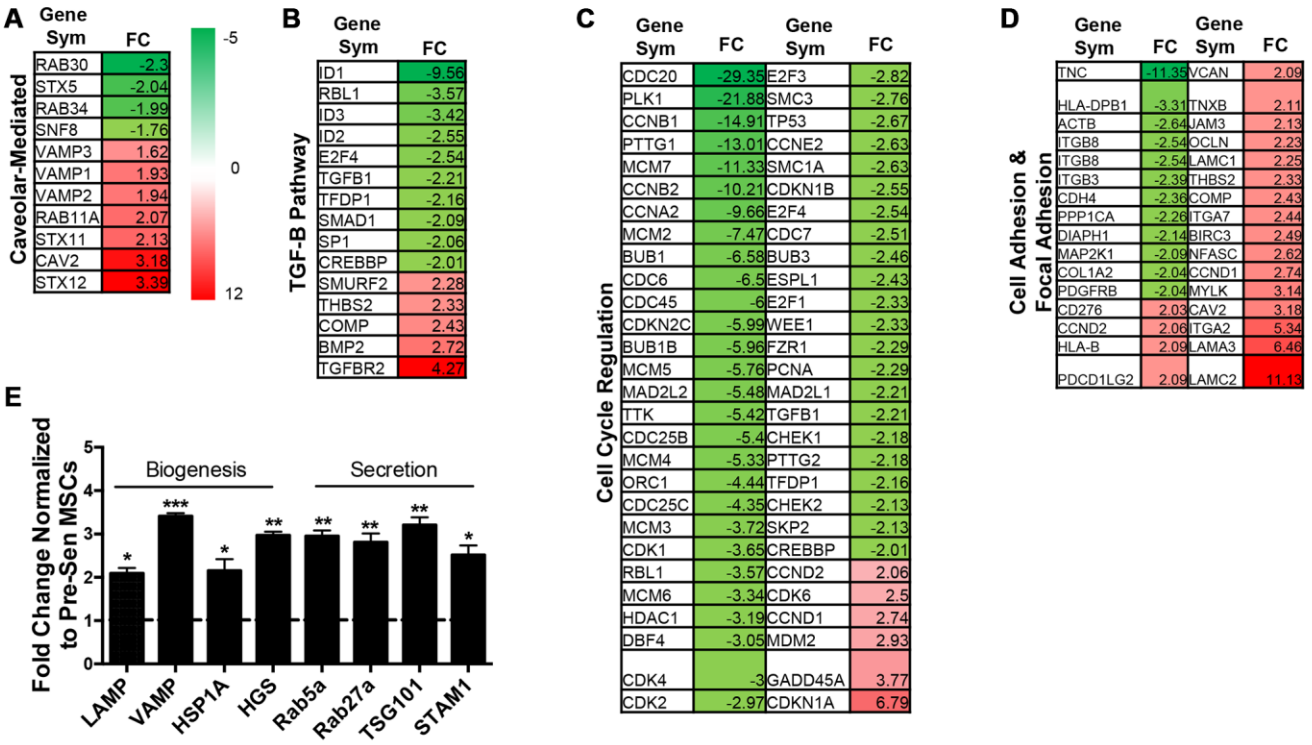

Supplementary Figure 1. Transcriptome fold changes of senescent MSCs vs. pre-senescent MCSs for pathways of (**A**) caveolar-mediated, (**B**) TGF-β, (**C**) Cell Cycle, and (**D**) Cell Adhesion and Focal Adhesion without FDR cutoff. All transcripts were significant $p < 0.05$. Gene expression in pre and senescent MSCs relating to EXO biogenesis and secretion pathways were examined using qRT-PCR. (**E**) Samples were normalized to β-actin and pre-senescent values (represented by black dashed line). $^{*}p < 0.05$, $^{**}p < 0.005$.

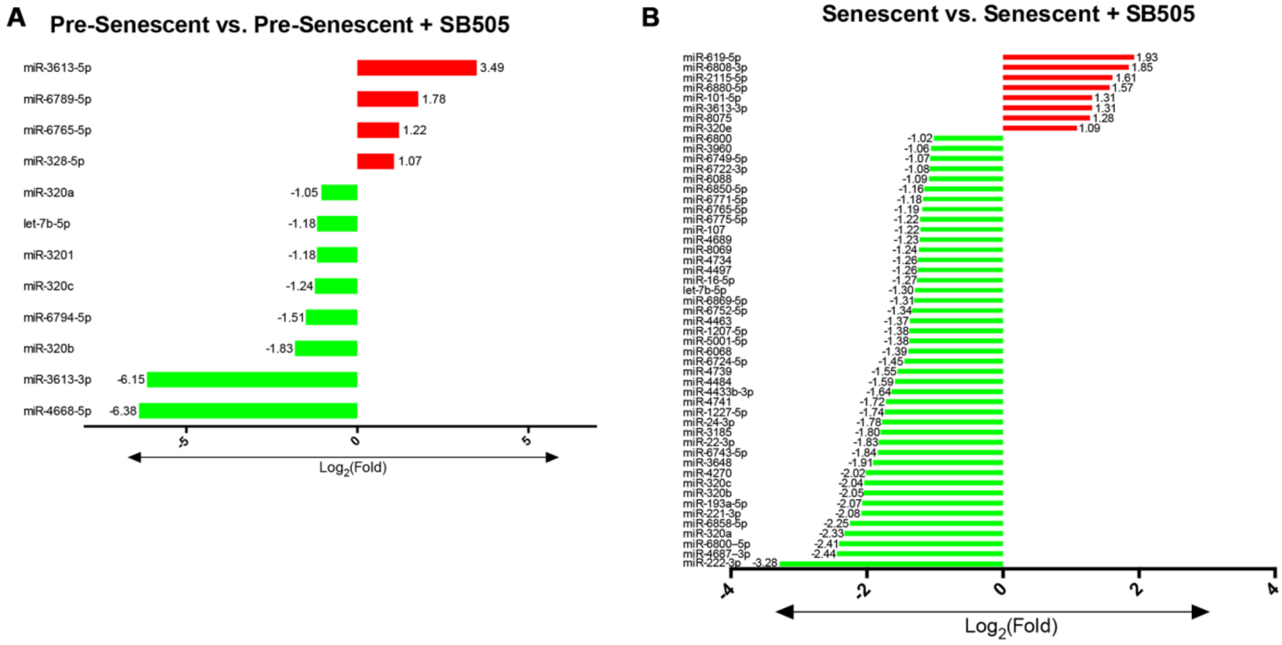

Supplementary Figure 2. A complete list of significantly altered EXO miRNAs (for Figure 5F, 5G) that were harvested from pre- and senescent MSCs that were treated with or without SB505 (**A**, **B**).

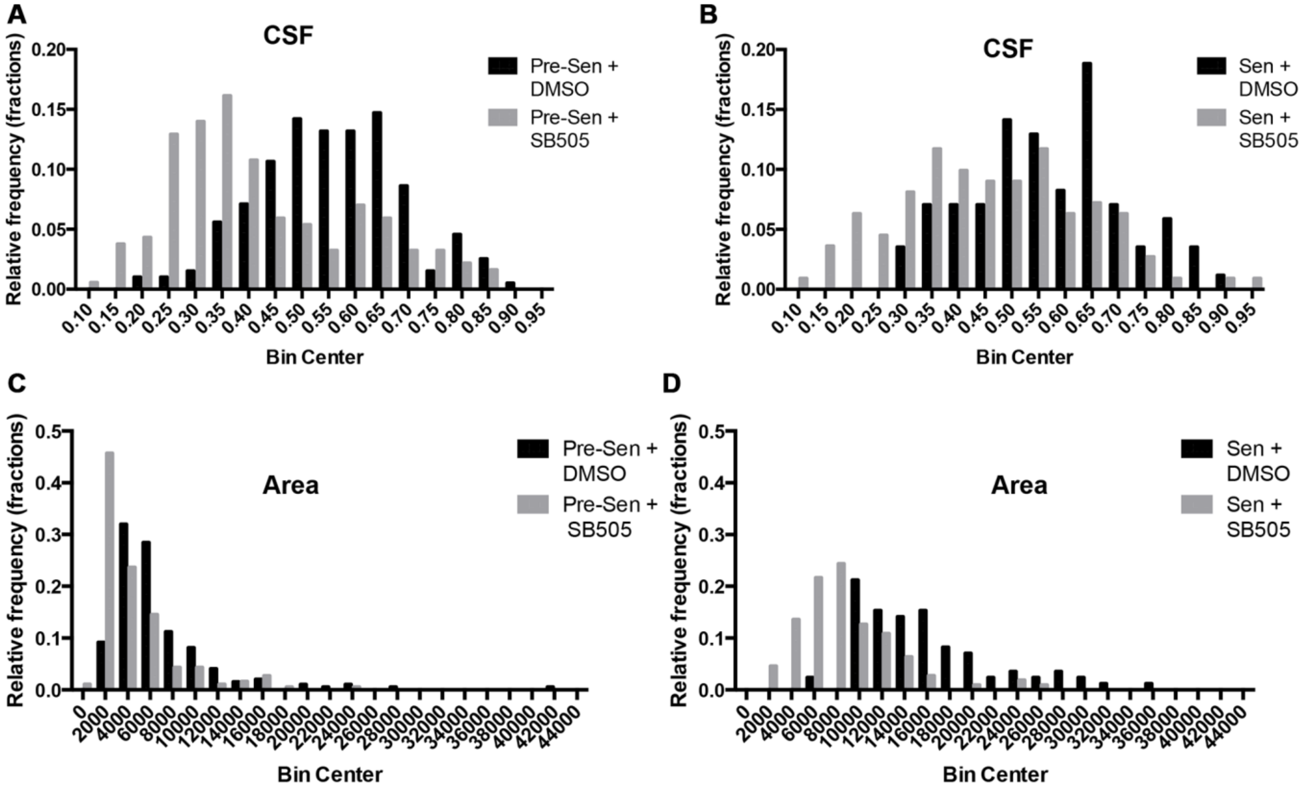

E

Coeff of Var	CSF	Area
**Pre-Sen**	0.24	0.71
**Pre-Sen + SB505**	0.42	0.83
**Sen**	0.25	0.39
**Sen + SB505**	0.38	0.51

Supplementary Figure 3. Histogram frequency distribution of cell shape factors (CSF) (**A**, **B**) and cell areas (**C**, **D**) of pre and senescent MSCs treated with SB505 (black) or DMSO control (gray). CSF and area values were reduced with SB505 treatment. The coefficient of variance (CoV) for CSF and area increased with SB505 compared to control groups, indicating that this treatment leads to increased heterogeneity in cellular morphology and area (**E**). The simultaneous decrease in cell area and increase in CoV values could correspond to a reduced number of cells developing senescence after SB505 treatment because we showed that senescent MSCs exhibit greater cell areas.

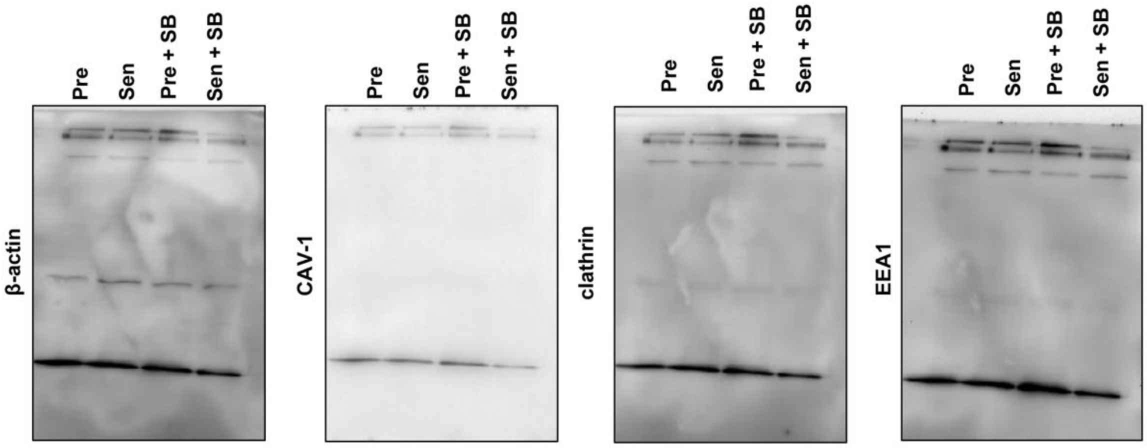

Supplementary Figure 4. (Full immunoblots) immunoblot exposures of EXO surface markers used in manuscript.
