# Supplementary material for: Senescence-associated exosomes transfer miRNA-induced fibrosis to neighboring cells
Source: Aging (Albany NY). 2023 Feb 23;15(5):1237–56. doi: 10.18632/aging.204539 (PMC10042705; doi:10.18632/aging.204539)
Supplement: Supplementary Table 1 [file aging-15-204539-s002.pdf]

SUPPLEMENTARY TABLE

Supplementary Table 1. Forward and reverse primer sequences used in manuscript.

**Primers**	
TGF-β	Forward: CAC CGG AGT TGT GCG GT<br>Reverse: GGC CGG TAG TGA ACC CGT TGA TG
TGF-βR1	Forward: TCT GCC ACA ACC GCA CTG TCA<br>Reverse: GGT AAA CCT GAG CCA GAA CCT GAC G
SMAD3	Forward: TGG ACG CAG GTT CTC CAA<br>Reverse: CCG GCT CGC AGT AGG TAA C
SMAD4	Forward: CTC ATG TGA TCT ATG CCC GTC<br>Reverse: AGG TGA TAC AAC TCG TTC GTA GT
LAMP-1	Forward: TCT CAG TGA ACT ACG ACA CCA<br>Reverse: AGT GTA TGT CCT CTT CCA AAA GC
VAMP-7	Forward: GAG GTT CCA GAC TAC TTA CGG T<br>Reverse: GAC ACT TGA GAA CTC GCT ATT CA
HSP1A	Forward: TTT GAG GGC ATC GAC TTC TAC A<br>Reverse: CCA GGA CCA GGT CGT GAA TC
HGS	Forward: CTC CTG TTG GAG ACA GAT TGG G<br>Reverse: GTG TGG GTT CTT GTC GTT GAC
Rab5a	Forward: AGA CCC AAC GGG CCA AAT AC<br>Reverse: GCC CCA ATG GTA CTC TCT TGA A
Rab27a	Forward: GCT TTG GGA GAC TCT GGT GTA<br>Reverse: TCA ATF CCC ACT GTT GTG ATA AA
TSG101	Forward: GAG AGC CAG CTC AAG AAA ATG G<br>Reverse: TGA GGT TCA TTA GTT CCC TGG A
STAM1	Forward: AAT CCC TTC GAT CAG GAT GTT GA<br>Reverse: CGA GAC TGA CCA ACT TTA TCA CA
